# Impact of a Pharmacist-Led Diabetes Management Intervention to Improve Health Equity

**DOI:** 10.1093/geroni/igab046.913

**Published:** 2021-12-17

**Authors:** Renae Smith-Ray, Tanya Singh, Evie Makris, Jaime Horan, Michael Taitel

**Affiliations:** 1 Walgreen Co, Deerfield, Illinois, United States; 2 Walgreens Co., Deerfield, Illinois, United States; 3 Walgreens, Deerfield, Illinois, United States

## Abstract

The COVID-19 pandemic and Black Lives Matter movement brought increased recognition to the need for health equity. Diabetes, the 7th leading cause of death, is one of many conditions where health inequities are evident. A higher percentage of Black (11.7%) and Hispanic (12.5%) U.S. adults are diagnosed with diabetes compared to non-Hispanic Whites (7.5%). To address this health inequity, a nationwide pharmacy chain implemented telephonic ‘Advanced Care’ (AC) outreach for patients with diabetes. During the AC call, pharmacists used motivational interviewing techniques to counsel patients on the importance of closing gaps in care and reducing barriers to medication adherence. Gaps included timely A1C testing, exams (eye, foot, kidney), immunizations (influenza, pneumonia, Hepatitis B), and recommendation of additional therapies for patients with multiple chronic conditions (ACE/ARB, statins). Medication fill gaps were compared between the Intervention period (8/1/20-1/31/-21) and a pre-intervention period (2/1/20-7/31/20). The AC pilot occurred in 8 Chicago Walgreens locations that primarily serve Black and Hispanic patients. Eight control stores were matched on census block-level household income and race/ethnicity, patient volume, and insurance mix. A pre/post-test vs. control difference-in-difference (DID) analysis was conducted to compare on-time refill rates. Of the 1,009 older patients (age≥50) called, 59.9% were reached. The DID analysis showed that patients in pilot stores had improved pre-post on-time refill rates compared to controls (p<0.0001). Diabetes self-management is key to reducing diabetes-related complications. Early findings from this pilot demonstrate that the Walgreens AC intervention improves medication adherence - an important step toward improving health equity.

